# Sensitivity and Specificity of Elliptical Modeling and Sagittal Lumbar Alignment Variables in Normal vs. Acute Low Back Pain Patients: Does Pelvic Morphology Explain Group Lordotic Differences?

**DOI:** 10.3390/healthcare13233163

**Published:** 2025-12-03

**Authors:** Paul A. Oakley, Ibrahim M. Moustafa, Joseph W. Betz, Jason O. Jaeger, Deed E. Harrison

**Affiliations:** 1 Independent Researcher, Newmarket, ON L3Y 8Y8, Canada; docoakley.icc@gmail.com; 2 Department of Physiotherapy, College of Health Sciences, University of Sharjah, Sharjah 27272, United Arab Emirates; iabuamr@sharjah.ac.ae; 3 Neuromusculoskeletal Rehabilitation Research Group, RIMHS–Research Institute of Medical and Health Sciences, University of Sharjah, Sharjah 27272, United Arab Emirates; 4 Independent Researcher, Boise, ID 83686, USA; drjoebetz@gmail.com; 5 Community Based Internship Program, Associate Faculty, Southern California University of Health Sciences, Whittier, CA 90604, USA; drjaeger@alianteipm.com; 6 Chiropractic Biophysics NonProfit, Inc., Eagle, ID 83616, USA

**Keywords:** acute low back pain, imaging, pelvic morphology, spine model, radiography, lumbar lordosis

## Abstract

**Background/Objectives:** The lumbar lordosis (LL) is influenced by pelvic morphology, the unique dimensional characteristics of the pelvis. We investigated the sensitivity and specificity of lumbar sagittal radiographic alignment variables to discriminate between normal controls and acute low back pain (ALBP) patients. **Methods:** A total of 50 normal controls (29 men; mean age of 27.7 ± 8.5 years) with no history of low back pain and 50 ALBP patients (29 men; 28.1 ± 8 years of age) were compared. Radiographic variables included three measures of LL, a b/a elliptical modeling ratio, sacral base angle (SBA), S1 posterior tangent to vertical (PTS1), two measures of pelvic morphology, as well as three relationships between morphology and LL. Descriptive statistics, linear correlations, and receiver operating characteristic (ROC) curves were calculated. **Results:** The SBA and Cobb T12-S1 LL were significantly greater in the ALBP group. The SBA showed a reasonable ability to discriminate between the normal and ALBP groups with ROC curve analysis (AUC = 0.67, optimal cut-off value = 41.2°, sensitivity = 0.70, and specificity = 0.62). Pelvic morphology was similar between sex and pain groups. **Conclusions:** Our sample of ALBP patients had similar pelvic morphology as compared to normal control participants; however, they also demonstrated an increased T12-S1 lordosis and sacral base angle, shown as a hyperlordosis.

## 1. Introduction

Even though the alignment of the lumbar lordosis (LL) is a human evolutionary adaptation to locomotion in the upright position [1], there is no consensus regarding the overall shape and magnitude of what represents a normal lordosis; this goal remains a challenge. The obstacles for determining a normative value for LL include the following: (1) the variability in analytical methods for quantification of lordosis, (2) radiographic positioning procedures, (3) race or geographic origin of the individual, (4) pediatric and advanced age, (5) sex, (6) body mass index, and (7) pelvic morphology [2,3,4,5,6].

Regardless of the discussed obstacles, many authors have proposed adult normative values of the LL [2,3,4,5,6]. In general, there have been large ranges of normative values for the LL reported in the literature; however, there are different measurement methods existing throughout the spine literature as well [3]. Using the Harrison posterior tangent method with a small standard error of measurement (SEM), it was demonstrated that normal control and pain populations could be differentiated statistically [7] and that the separation in degrees is small; thus, the measures having the smallest SEM values should be used. Traditionally, however, the 4-line Cobb angle of measurement for LL, with a relatively high SEM, has been the standard and has possibly contributed to confusion regarding the inability to differentiate back pain populations from normal controls [8].

Importantly, the LL for a given individual may be affected by the patient’s specific pelvic morphology [9], which is essentially the structural and dimensional characteristics of the pelvis, including the interrelated parameters of pelvic incidence (PI), pelvic tilt (PT), and sacral slope (SS) (i.e., PI = PT + SS). Thus, both the morphology and measurement used to assess LL may affect the outcome of abnormal vs. normal alignment relative to the specific patient with low back pain (LBP) [10]. In a recent study, for example, it was determined that pelvic morphology parameters were not different between an asymptomatic control group compared to a chronic low back pain (CLBP) patient population [10]. However, the CLBP group had an abnormal ‘fit’ of their LL relative to their pelvic morphology compared to the fit of the normal group; that is, whether a steeper sacral slope is matched to a greater lordosis, and vice versa [10]. Thus, assessing both the LL and the corresponding pelvic parameters is important in the screening of patients with CLBP, and this can only be performed via routine X-ray screening.

Regarding the assessment of acute LBP (ALBP), it is generally understood that for uncomplicated, spine-related ALBP, initial radiographic imaging is not necessary [11,12,13,14,15,16,17]. The reasons for this logic are generally rationalized as follows [18]: 1. radiographs usually do not reveal the ‘cause’ of the pain to warrant a specific treatment (i.e., low diagnostic yield); 2. red flag medical conditions are very rare (e.g., malignancies, etc.); and, lastly, 3. exposure to radiation and its ‘carcinogenic effect’ is considered unnecessary. First, and in contrast to the ‘popular’ opinion, low-dose radiation exposures via spinal X-rays are considered negligible, and there is now strong scientific evidence disproving the false flag ideology that spine X-rays can cause cancer [19,20,21,22,23,24]. Next, although medical red flag conditions are considered to be rare in occurrence [25], for those who actually have a medical condition that would not have been otherwise detected, it immediately becomes a critically important factor in their treatment triage [26]. Finally, what if differences in biomechanical alignment parameters were detectable in those with ALBP and could dictate or modify specific treatment considerations? It is this last scenario that we aim to explore in this study. The purpose of this investigation is to explore the differences in a group of carefully screened asymptomatic participants to a group of demographically matched, first-time mechanical ALBP patients, specifically comparing standard biomechanical X-ray parameters of both lumbar curve and pelvic morphology values. We hypothesize that there will be biomechanical differences based on X-ray parameter measurements between the groups.

## 2. Methods

### 2.1. Patient Data

In a previous study, radiographic data from 4 matched groups were compared in terms of elliptical modeling of the LL [7]. Herein, we revisit the data from 2 of the groups, the asymptomatic control group (n = 50) [3] and the ALBP group (n = 50) [7]. Thus, this is a retrospective review of clinical records from the previous investigations and is, therefore, exempt from IRB approval under section 45 CFR 46.101(b)(4). See https://www.hhs.gov/ohrp/regulations-and-policy/decision-charts-pre-2018/index.html#c5 (Accessed on 10 April 2024).

The normal group consisted of 50 participants (29 male/21 female) with an average age of 27.7 years, height of 171.5 cm, and weight of 70.8 kg. These participants were consecutively chosen who demonstrated a normal physical exam, reported no history of back pain, and were screened to be free of any bony abnormalities (i.e., pathologies, anomalies, instability, or degeneration) as visualized on lateral lumbar radiographs. The ALBP group consisted of 50 patients who first reported an ‘acute’ LBP as the first ever episode of mechanical LBP that had been present for a period of less than 6 weeks. The diagnosis for a mechanical source of LBP was determined after appropriate patient history, physical examination, and diagnostic imaging, which ruled out other sources such as red flags, chronic conditions, or the presence of lumbar scoliosis > 10°. The ALBP group was selected to match the control group, thus having an equal male/female ratio (29/21), an average age of 28.1 years, height of 174 cm, and weight of 74 kg. The ALBP group was selected randomly from patient files who met the specific inclusion criteria.

Radiographs were taken with the patient standing barefoot with the right side to the grid cabinet. To keep the weight of the upper extremities as close to the midaxillary line as in the standing position, the arms were positioned so the hands were placed on the head with the arms held laterally in the coronal plane [3].

### 2.2. Lumbar Modeling

As described elsewhere [7,27], all patient radiographs were digitized with a sonic digitizer (GP-9, Science Accessories Corp., Shelton, CT, USA). Eighteen points, including the vertebral body corners from the posterior-inferior T12 to the posterior-inferior S1, as well as the superior aspect of each femur head, pubic symphysis, the anterior-inferior corner of T12, and the anterior-superior corner of S1, were digitized on the lateral lumbar radiographs. These points were stored as x-y points, where an original computer code was used to run a least squares approximation of each subject’s LL in the shape of an ellipse. Specifically, the program iterates to determine the best-fit ellipse from T12-S1, and from this, a semi-major (a) and semi-minor (b) axis are determined as the b/a ratio, which represents the portion of a quadrant (between 80° and 90°) that the elliptical LL represents [7,27].

### 2.3. Radiographic Measurements

**Figure 1:**
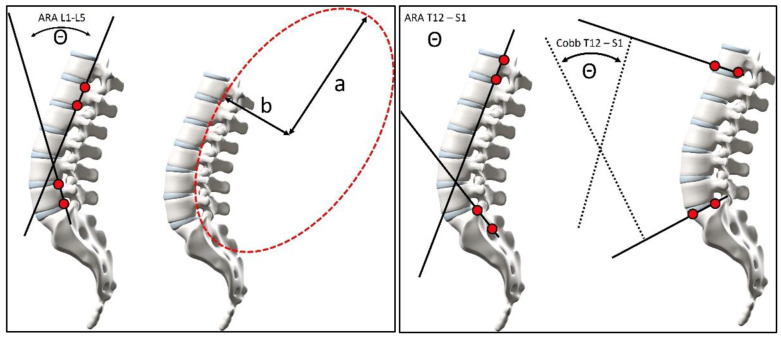
Left to right: Absolute rotation angle (ARA) of L1-L5; b/a ratio; ARA T12-S1; and Cobb angle of inferior T12 to superior S1.

**Figure 2:**
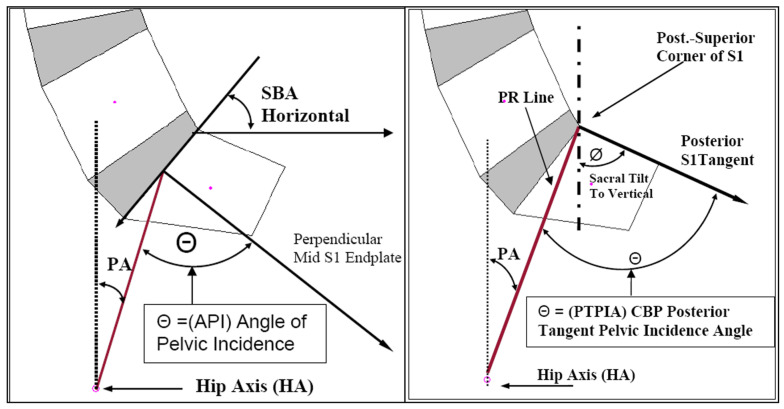
Left: The angle of pelvic incidence (API) of Legaye et al. [30]. The angle Θ between the perpendicular mid S1 endplate line and the hip axis (HA) mid S1 body line is termed the angle of pelvic incidence. Two additional angles are shown: the sacral base to the horizontal angle (SBA) and the pelvic tilt angle (PA) measured from the mid-S1 body to the hip axis line relative to a vertical line originating at HA. Right: posterior tangent pelvic incidence angle (PTPIA) from Harrison et al. [32] is the angle Θ between the PR line and the S1 posterior tangent line. Two additional angles, PA and sacral tilt to the vertical, are shown. The angle PA is constructed between the pelvic radius (PR) line and a line drawn vertically through the hip axis. The sacral tilt angle is constructed between the S1 posterior tangent line and the vertical.

The radiographic parameters acquired for this study were calculated from the raw x-y coordinates from the assembled digitized X-rays. Standard measures included global lordosis angles as determined from the posterior tangents of L1 and L5 (absolute rotation angle, ARA L1-L5), a modified version of this angle extending from ARA T12-S1, as well as the Cobb T12-S1, or the angle formed between the inferior endplate of T12 and the superior endplate of S1 (Figure 1). The sacral base angle (SBA) was determined by the angle formed between the superior endplate of S1 and the horizontal, and the S1 posterior tangent line relative to the vertical (PT-S1) was also determined (Figure 2). A negative value indicates spinal extension or lordosis. These measurements have been shown to be reliable, having a standard error of measurement of about 2° [28,29].

Two unique measurements were also made that summarize relationships related to pelvic morphology (Figure 2): the angle of pelvic incidence (API) and the posterior tangent pelvic incidence angle (PTPIA). The API uses the method of Legaye et al. [30] and represents an alternate method from During et al. [31]. The second pelvic morphology angle is Harrison et al.’s [32] PTPIA, as a representation of the pelvic morphology being consistent with the posterior vertebral body tangent method of lumbar lordosis analysis. Thus, the PTPIA uses the posterior body of S1, while the API method uses the SBA in construction; each pelvic morphology measure may represent different correlations and applications. Also, as suggested by Diebo et al. [33], we assessed the relationship of the variables of lumbar lordosis subtracted from the API.

### 2.4. Statistical Analysis

The means, standard deviations, maximum, and minimum values for the demographic and radiographic variables between the groups were analyzed. The Shapiro–Wilk and Kolmogorov–Smirnov normality tests were used to assess the distribution of the variables, and since several were nonparametric, Spearman’s rank correlation coefficient was used to assess the correlations between the variables of interest. Linear correlation coefficients were investigated between the continuous variables for both pain groups (normal and ALBP). The correlations investigated included the correlation between pelvic morphology variables (API and PTPIA) and the LL, sacral tilt, and elliptical ratio b/a variables.

Receiver operating characteristic curves (ROC curves) were used to evaluate the sensitivity and specificity of alignment variables and their ability to classify between the pain groups. Pelvic morphology, lumbar lordotic angles, sacral angles, elliptical b/a ratio, API-ARA T12-S1, API-ARA L1-L5, and API-Cobb T12-S1 were all investigated in the classifier models. For each ROC curve, the area under the curve (AUC), optimum cutoff value between groups, and sensitivity and specificity thresholds were reported. Criterion for determining the optimal cutoff was the maximum value of Youden’s index and Kolmogorov—Smirnov (K-S) metric. The largest reported value is used in cases of multiple cutoff values associated with the maximum K-S metric. For all statistics, SPSS (v.29) was used (IBM Corp., Armonk, NY, USA).

Depending on the results of the findings, any statistically significant radiographic parameter variables that emerge between the controls and ALBP patients were investigated as to the validity of making statistical claims of these differences by determining a post hoc sample size requirement. We performed this by first calculating the Cohen’s d effect size apparent from the data (https://www.psychometrica.de/effect_size.html (accessed on 10 April 2024), and then performing a post hoc sample size analysis (https://www.danielsoper.com/statcalc/calculator.aspx?id=47 (accessed on 10 April 2024).

## 3. Results

The means, standard deviations, maximums, and minimums for the control group (CG) and the ALBP groups are shown in Table 1. The only statistically significant differences between the groups showed the ALBP group had both a greater Cobb T12-S1 (−70° vs. −65.4°, *p* = 0.021) and sacral base angle (SBA) (44.5° vs. 39.4°, *p* = 0.003).

Correlation coefficients between the key variables for the CG and ALBP groups are shown in Table 2. Notably, the API was positively correlated with the SBA in both groups (CG: rho = 0.492, *p* < 0.001; ALBP: rho = 0.658, *p* < 0.001), negatively correlated with the absolute rotation angle of LL from L1-L5 (ARA L1-L5) in the control (rho = −0.325, *p* = 0.021), and negatively correlated with Cobb T12-S1 in the ALBP group (rho = −0.539, *p* < 0.001). The PTPIA positively correlated with the posterior tangent of S1 to the vertical (PTS1) in both groups (CG: rho = 0.504, *p* < 0.001; ALBP: rho = 0.628, *p* < 0.001) and negatively correlated with the ARA L1-L5 in the control group (rho = −0.305, *p* = 0.031), and also negatively correlated with ARA T12-S1 in the ALBP group (rho = −0.465, *p* < 0.001). The b/a elliptical LL model ratio was negatively correlated with all the LL values for both groups (CG: rho = −0.31–0.38, *p* < 0.03; ALBP: rho = −0.36–0.61, *p* < 0.011). The SBA was negatively correlated with all LL measures in both groups (CG: rho = −0.59–0.73, *p* < 0.001; ALBP: rho = −0.32–0.86, *p* < 0.022), but showed to be strongest for the Cobb T12-S1 measure (CG: rho = −0.731, *p* < 0.001; ALBP: rho = −0.862, *p* < 0.001).

**Figure 3:**
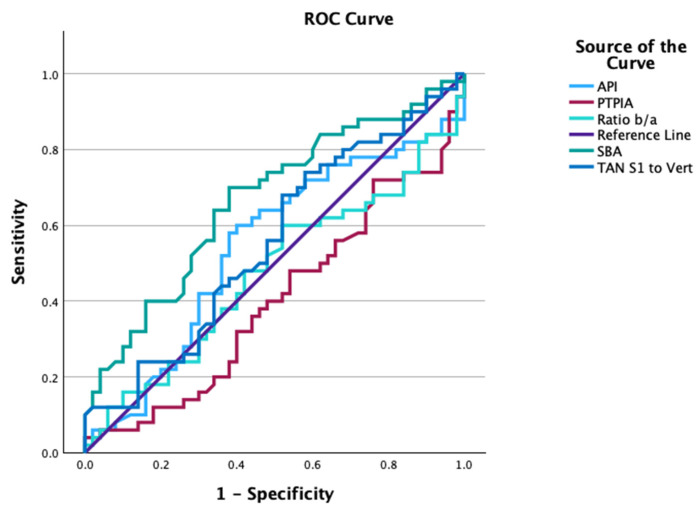
Receiver operating characteristic (ROC) plots for API, PTPIA, Ratio b/a, SBA, and PT-S1 (TAN S1 to Vert). A variable that could distinguish between normal and ALBP groups was the SBA (green): area under the curve (AUC) = 0.665, optimal cut-off value = 41.2°, sensitivity = 0.70, and specificity = 0.62.

**Figure 4:**
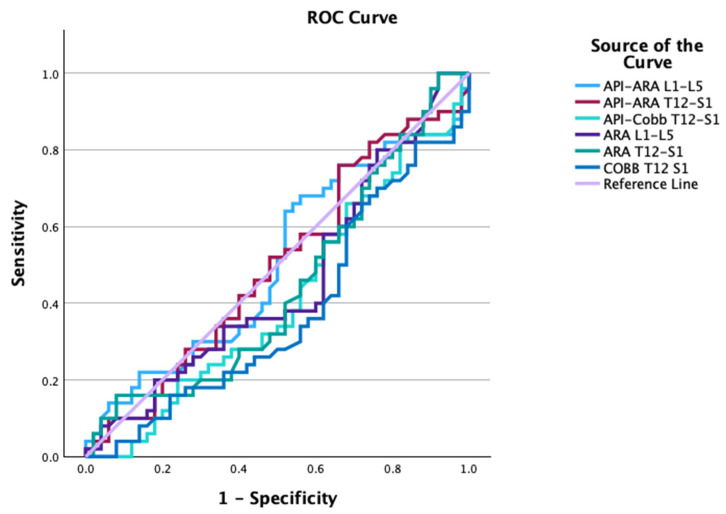
Receiver operating characteristic (ROC) plots for API-ARA L1-L5, API-ARA T12-S1, and API-Cobb T12-S1, as well as global lordosis measures of ARA L1-L5, ARA T12-S1, and Cobb T12-S1. None of the parameters of API—lumbar lordosis values (ARA L1-L5, ARA T12-S1, and Cobb T12-S1) or lumbar lordosis curve measures (ARA L1-L5, ARA T12-S1, and Cobb T12-S1) demonstrated good classification ability between controls and ALBP groups.

The results of the receiver operating characteristic (ROC) curves are shown in Table 3, Figure 3 and Figure 4. Table 3 shows the analyses of the area under the curve (AUC), the optimum cutoff, and the sensitivity and specificity at this cutoff for the ROC curves. Notably, the SBA showed a reasonable ability to discriminate between the normal and ALBP groups (AUC = 0.67, optimal cut-off value = 41.2°, sensitivity = 0.70, and specificity = 0.62). Figure 3 shows the ROC curve plots for API, PTPIA, elliptical ratio b/a, SBA, and PT-S1. Figure 4 shows the ROC curve plots for API-ARA L1-L5, API-ARA T12-S1, and API-Cobb T12-S1, as well as the LL measures of ARA L1-L5, ARA T12-S1, and Cobb T12-S1.

**Figure 5:**
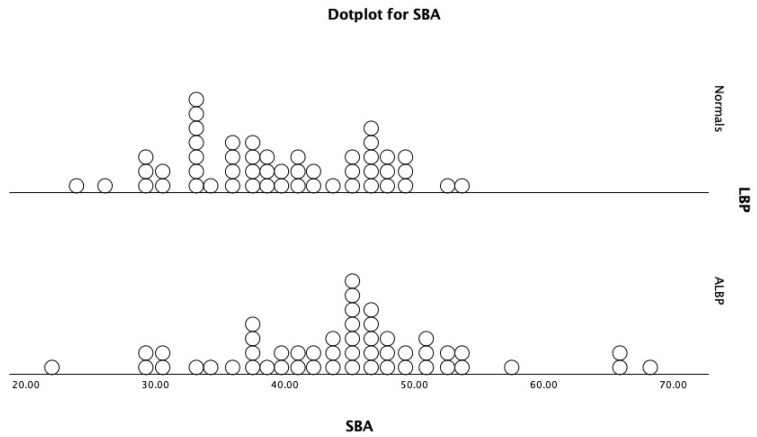
Dotplot illustrating the relationship of sacral base angle (SBA) for normal and acute low back pain (ALBP) groups. The top dotplot is the normal control group, and the bottom dotplot is the ALBP group. SBA increases moving to the right and decreases moving to the left. Patients with ALBP tend to have greater SBA (44.5° vs. 39.4°, *p* = 0.003), and the optimal cut-off value = 41.2°.

**Figure 6:**
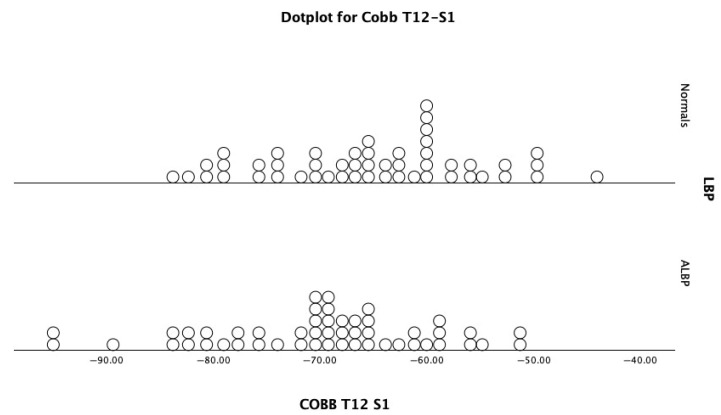
Dotplot illustrating the relationship of the lumbar lordosis Cobb T12 to S1 for normal and acute low back pain (ALBP) groups. The top dotplot is the normal control group, and the bottom dotplot is the ALBP group. Cobb T12-S1 increases moving to the left and decreases moving to the right. Patients with ALBP tend to have greater lordosis values (−70° vs. −65.4°, *p* = 0.021).

**Figure 7:**
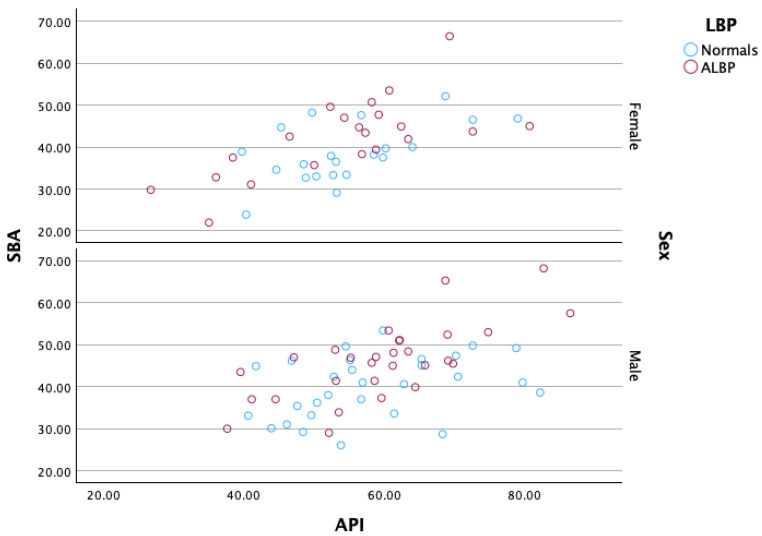
Sacral base angle (SBA) vs. pelvic morphology (API) partitioned by pain level (controls ‘Normals’ vs. acute low back pain ‘ALBP’). The scatterplot on the top is for females, and on the bottom for males. The color codes are as follows: blue for normals, red for ALBP patients. Collectively, these graphs show that the range of values of both SBA and API is smaller for normal compared to ALBP patients. Apart from this, there are no other apparent differences in the SBA vs. API relationship for either pain category or sex.

Figure 5 and Figure 6 show dotplots of the data for the controls and ALBP groups for the SBA and Cobb T12-S1, respectively. Figure 7 displays a scatterplot of the SBA vs. the API for both males and females. The distribution of both the SBA to horizontal and pelvic morphology variables was narrower for the CG compared to the ALBP group; however, no difference in the SBA vs. pelvic morphology relationships for either pain category or sex was found.

## 4. Discussion

In this study, it has been shown that the SBA and lumbar lordosis (Cobb T12-S1) revealed increased magnitudes in ALBP patients versus healthy controls. Likely due to sample size limitations, only the SBA showed reasonable ability to distinguish between the ALBP and normal groups in binary classifier modeling. Importantly, results for males and females were similar, showing that pelvic morphology does not significantly differ between normal asymptomatic controls and ALBP patients. Therefore, the differences between groups are likely due to the differences in how the SBA fits to the lumbar lordosis, given a specific morphological profile of the pelvis. The findings support our original hypothesis that certain radiographic variables would be able to differentiate ALBP patients from carefully screened asymptomatic healthy control participants without LBP.

Our analysis was based on a previously available dataset [7], and this publication lacked more sophisticated analysis of data, including no measurements of pelvic morphology, no analysis of correlation between key variables, and no ROC analysis for sensitivity and specificity of cutoff values; this was a primary reason we chose to reanalyze this data. Furthermore, this previous publication [7] lacked a sample size power analysis. Due to this constraint, herein, we performed a post hoc effect size calculation for the SBA from our data analysis and found it to be 0.617; in turn, a power calculation determined a sample size of 90 participants (45 per group) necessary. Therefore, our sample (n = 100) would seem adequate to make statistical claims regarding the SBA, and this is likely why the SBA variable emerged to be an adequate classifier in the ROC curve analysis. Alternatively, a post hoc effect size calculation for the Cobb T12-S1 was 0.471, and the power calculation indicated a sample size of 144 participants (72 per group) was necessary. Thus, despite the Cobb T12-S1 showing a statistically larger magnitude in the ALBP group, it failed to emerge as an adequate classifier in the ROC curve analysis, as it was underpowered by the sample size.

The main finding of increased SBA, indicative of a forward-rotated pelvis and increased lumbar lordosis in ALBP subjects versus healthy controls, indicates that there are biomechanical variables that differ between these cohorts. Thus, the rationale to perform radiographic assessment of a patient experiencing a first-time occurrence of a mechanical LBP episode seems logical, particularly as external contour measures for assessing low back alignment have reliability issues and do not correlate well with true radiographic spine alignment [35,36]. This finding, however, is in direct opposition and is contrary to current LBP guidelines, which recommend against routine imaging for ALBP [11,12,13,14,15,16,17]. Although the traditional perception of radiographic imaging for LBP (including chronic) has been that it does not aid in the diagnosis and treatment of LBP, this perception has been a perpetuated myth for decades [37,38,39,40,41,42,43]. Two recent systematic reviews (SRs) have determined that radiographic imaging can show important biomechanical parameters and other radiographic findings that are statistically associated with and predictive of LBP [38,39]. While the Chun et al. [38] SR was specific to CLBP and identified that a hypolordosis of the LL was strongly correlated to CLBP, the SR from Raastad and colleagues [39] was not specific to CLBP, and they identified that disk space narrowing, osteophytes, spondylosis, and spondylolisthesis were all linked to the presence of LBP, whether acute or chronic.

The findings of this study, although needing further verification by continued investigation, point to another piece of evidence showing a theme of flawed dated ideology associated with delayed imaging of first-time ALBP patients, and our work is not inconsistent with other SRs in the literature [40]. For example, Sadler and colleagues [40] identified that an altered lumbar lordosis was statistically linked to the development of LBP, requiring intervention.

Reigo et al. [41] determined that a flattened, altered lumbar lordosis was prospectively associated with new and long-term sick leave resulting from LBP in a group of 383 participants aged 20–59. Steinberg et al. [42] determined that half of a sample of 464 male, age-matched (18 years ± 2 months) army recruits had a history of LBP. Those having LBP histories included the more frequent X-ray findings of right-sided scoliosis, abnormal lumbar lordosis, sacral or lumbarization, wedge vertebra, bilateral spondylolysis of L5, and/or a sagittal central canal diameter of less than 12 mm. They concluded that “Given that radiographic screening shows that LBP is more common in those with spinal deformity it may be a reasonable means of predicting which individuals are more likely to develop LBP” [42]. Finally, Reinert [43] identified a correlation of the location of pain with altered alignment positions of the lumbar spine in 110 patients presenting with ALBP.

As indicated above, the ideology that radiographic imaging is inappropriate for ALBP has a considerable history and evidence in the spinal literature [11,12,13,14,15,16,17,44]. However, oftentimes, the interpretation of some of this data has been overstated and not placed in the proper context [11,18,19]. For instance, Gushcha et al. [11] emphatically stated the following: “In fact, routine imaging for acute LBP can actually have a negative effect as it may reveal incidental radiographic findings that exacerbate patient fear and anxiety”, while simultaneously acknowledging the weakness of the evidence in support of their statement. As another example, while advocating against imaging for the ALBP patient, the Kerry et al. (2002) study [14] identified patients receiving X-rays of their lower backs had better psychological well-being and lower depression scores at 1-year follow-up, which seems counter to the authors’ own conclusions.

Likewise, it was determined in the Kendrick et al. studies [13,44] that patients who received X-rays of their low back were more satisfied with the care received. Further, participants who were randomized to a preference group, where the choice for using X-rays was made by the patient, achieved better outcomes versus those randomized to a group where the choice was not theirs [44]. Ironically, abnormal findings were identified on two-thirds of the X-rays and at the 9-month follow-up, 61% of patients remained in pain.

It is important to note that the diagnosis of true ALBP is generally overutilized, where investigations have identified that up to 76% of patients with ALBP actually have a recurrent history of ‘acute’ flare-ups [45]. This would indicate that the majority of adults with ALBP actually have a chronic condition with episodes of acute pain or exacerbations. Furthermore, this is supported by the fact that many patients seeking primary care for acute or new episodes of LBP continue to report disability several years after their initial visit and treatment [13,14,46]. Still, the question remains whether or not there is a biomechanical component both to the first-time occurrence of ALBP and its continued ‘acute’ flare-up; our data indicates that in our sample of patients, there seems to be, in part, an altered sagittal plane curvature. A second question arises regarding the proper treatment approaches based on the unique radiographic findings of the patient and their individual needs. While this question needs to be continually investigated in high-quality trials, there is preliminary evidence in the form of randomized trials [47] and case reports [48] demonstrating successful reduction in lumbar hyperlordosis and improvement of function using specific exercises and myofascial work [47] as well as lumbo-pelvic curve reducing traction methods [48].

An important implication associated with routine imaging of ALBP patients is the concern over radiation exposures. There are many recent overviews of why the traditional radiophobic view towards diagnostic radiation is erroneous [18,19,20,21,22,23,24,26]. The model used for cancer risk assessment is the linear no-threshold (LNT) assumption, which assumes a linear relationship between radiation exposure and genetic damage. From this, the ‘as low as reasonably achievable’ (ALARA) principle was born [19]. ALARA is used throughout medicine in an attempt to reduce/avoid radiologic imaging in order to reduce the presumed risk of inducing future malignancy. However, there are no valid data to support the use of the LNT model in the low-dose range (e.g., exposures received by obtaining spinal X-rays), so the dose as a surrogate for risk in radiological imaging is not appropriate, and, therefore, the use of the ALARA concept is obsolete [19]. Importantly, Calabrese [20,22] has shown that historical assessments of the events leading up to the original adoption of the LNT model for cancer risk assessment reveal that their scientific foundations were (and still are) inaccurate and its adoption was also unethical (see Calabrese [22]). Calabrese argues for a significant historical, scientific, and regulatory remediation of the unscientific use of the LNT model in current cancer risk assessment [20]. Thus, fear associated with taking spinal X-rays, in this case for ALBP patients, is certainly unjustified. Further, based on our results, delaying X-rays based on the ALARA principle may be unethical as the biomechanical information gained from imaging may lead to an evidence-based treatment strategy based on the unique imaging findings, which is at the heart of patient-centered care [47,48].

Limitations to this study include the limited sample size; this is also likely why the lumbar lordosis Cobb T12-S1 variable failed to reveal adequate classification ability in the ROC curve analysis. We were limited, however, as this was a re-analysis of a previously existing database. Further, because the average age of our sample was relatively young, the generalizability of these findings is limited to this age group. Another limitation is that data pertaining to pain intensity and disability, as well as lifestyle factors such as occupation, were not available; therefore, it is not possible to control or investigate how these variables may be related to the biomechanical radiographic parameters. A final limitation is that although we used biomechanical radiographic parameters, our measures were more global; however, studies have shown that other vertebral measures, such as intervertebral disk height, can distinguish LBP from healthy controls [49,50]; thus, it is unknown if these parameters are important in the distinction between ALBP patients and controls.

## 5. Conclusions

ALBP patients have an increased T12-S1 lumbar lordosis and sacral base angle, expressed as hyperlordosis. Pelvic morphology is similar between sex and pain groups, and, therefore, does not explain group differences. The differences between groups are likely due to the differences in how the SBA fits to the lumbar lordosis, given a specific morphological profile of the pelvis. Patients with a SBA greater than 41.2° have a high likelihood of displaying lumbar hyperlordosis amenable to contemporary treatments. Our findings are novel; however, if verified, would support routine imaging for ALBP patients in order to screen and diagnose biomechanical parameters that may lead to evidence-based patient-specific medical management.

## Figures and Tables

**Table 1:** Means, standard deviations (SD), maximum, and minimum values for all variables used in the analysis of normal and acute low back pain (ALBP) patients.

Variable	Normal Group			ALBP Patients			
	Mean ± SD	Max	Min	Mean ± SD	Max	Min	*p* Value
Age (yrs)	27.7 ± 8.5	52	18	28.1 ± 8	50	14	*p* > 0.05
Height (cm)	171.5 ^$^	^$^	^$^	174 ^$^	^$^	^$^	*p* > 0.05
Weight (kg)	70.8 ^$^	^$^	^$^	74 ^$^	^$^	^$^	*p* > 0.05
Sex	29 Males, 21 Females			29 Males, 21 Females			
ARA L1-L5	−40.2 ± 9.5°	−22.1°	−62.9°	−40.9 ± 9.1°	−14.4°	−55.9°	*p* = 0.718
ARA T12-S1	−76.3 ± 9.9°	−47.8°	−97.3°	−77.5 ± 9.4°	−51.4°	−91.8°	*p* = 0.562
Cobb T12-S1	−65.4 ± 9.4°	−44.2°	−83.4°	−70.0 ± 10.1°	−51.4°	−95.5°	*p* = 0.021 *
SBA	39.4 ± 7.2°	53.4°	23.9°	44.5 ± 9.2°	68.2°	22°	*p* = 0.003 **
PT-S1	50.3 ± 7.8°	62.1°	29.7°	51.9 ± 7.8°	67.9°	33.9°	*p* = 0.310
API	56.8 ± 11°	82.2°	40.6°	57.4 ± 12.5°	86.5°	35°	*p* = 0.815
PTPIA	73.9 ± 8.9°	92.9°	57.1°	70.6 ± 9.5°	93.7°	55.8°	*p* = 0.076 ^a^
b/a	0.389 ± 0.147	0.874	0.150	0.389 ± 0.23	1.5	0.027	*p* = 0.996
API–ARA T12-S1	−19.5 ± 14.4°	13.8°	−47.6°	−20.1 ± 15.4°	16.3°	−55.9°	*p* = 0.850
API–ARA L1-L5	16.6 ± 11.5°	40°	−7.8°	16.5 ± 13.6°	40.9°	−15.3°	*p* = 0.962
API–Cobb T12-S1	−8.6 ± 12.9°	18.9°	−33.7°	−12.6 ± 10.7°	8.3°	−31.3°	*p* = 0.092 ^a^

Note: Variables are as follows: (1) the intersection of posterior body lines at L1-L5 forming a global angle of the lumbar lordosis (ARA L1-L5), (2) the intersection of posterior body lines at T12-S1 forming a global angle of the lumbar lordosis (ARA T12-S1), (3) the intersection of the inferior endplate line of T12 and the superior endplate line of S1 forming a Cobb angle (Cobb T12-S1), (4) the superior sacral endplate line relative to the horizontal (SBA), (5) the posterior tangent of S1 relative to the vertical (PT-S1), (6) angle of pelvic incidence (API), (7) posterior tangent pelvic incidence angle (PTPIA), and (8) elliptical minor/major axis ratio (b/a). A negative value indicates spinal extension or lordosis. ^$^ Data is missing from original publication records, so information could not be retrieved. Independent samples two-sided *t*-test: * indicates *p* < 0.05; ** indicates *p* < 0.01; and ^a^ indicates approaching significance.

**Table 2:** The matrix of correlations separated by normal and acute low back pain groups and respective *p*-values for individual tests of significance that the correlation is zero vs. not zero.

Group	Variable	PTS1	ARA T12S1	Cobb T12S1	ARA L1L5	SBA	API	PTPIA
Normal								
	SBA	0.799 ** p < 0.001	−0.602 ** p < 0.001	−0.731 ** p < 0.001	−0.592 ** p < 0.001			
	API	0.248 *p* = 0.083	−0.059 *p* = 0.685	−0.215 *p* = 0.134	−0.325 * p = 0.021	0.492 ** p < 0.001		
	PTPIA	0.504 ** p < 0.001	−0.248 *p* = 0.082	−0.197 *p* = 0.171	−0.305 * p = 0.031	0.460 ** p < 0.001	0.850 ** p < 0.001	
	b/a	0.174 *p* = 0.228	−0.355 * p = 0.011	−0.596 ** p < 0.001	−0.609 ** p < 0.001	0.461 ** p < 0.001	0.195 *p* = 0.174	0.055 *p* = 0.704
Acute pain								
	SBA	0.329 * p = 0.020	−0.324 * p = 0.022	−0.862 ** p < 0.001	−0.469 ** p < 0.001			
	API	0.102 *p* = 0.482	−0.059 *p* = 0.685	−0.539 ** p < 0.001	−0.239 *p* = 0.095	0.658 ** p < 0.001		
	PTPIA	0.628 ** p < 0.001	−0.465 ** p < 0.001	−0.074 *p* = 0.611	−0.141 *p* = 0.330	0.108 *p* = 0.455	0.488 ** p < 0.001	
	b/a	0.054 *p* = 0.707	−0.334 * p = 0.018	−0.307 ** p = 0.030	−0.376 ** p = 0.007	0.143 *p* = 0.320	0.001 *p* = 0.997	0.052 *p* = 0.718

Note: Variables are as follows: posterior tangent S1 to vertical (PTS1), sacral base angle to horizontal (SBA), lumbar lordosis T12-S1 (ARA T12-S1 and Cobb T12-S1), lumbar lordosis L1-L5 (ARA L1-L5), angle of pelvic incidence (API), posterior tangent pelvic incidence angle (PTPIA), and elliptical minor/major axis ratio (b/a). Spearman’s rho, * *p* < 0.05, ** *p* < 0.01.

**Table 3:** Analyses of the area under the curve (AUC), the optimum cutoff, and the sensitivity and specificity at this cutoff for receiver operating characteristic curves.

Groups	Variable	AUC	Cutoff	Sensitivity	Specificity
Normal vs. Acute	ARA L1-L5	0.457	−56.2°	1.0	0.08
	ARA T12-S1	0.445	−66.8°	0.16	0.92
	Cobb T12-S1	0.374	−43.2°	0.0	0.0
	SBA	0.665	41.2°	0.70	0.62
	PT-S1	0.555	49.5°	0.68	0.48
	API	0.542	57.1°	0.58	0.62
	PTPIA	0.399	93.1°	0.04	0.0
	b/a	0.475	0.35	0.60	0.48
	API–ARA T12-S1	0.498	−27.6°	0.76	0.34
	API–ARA L1-L5	0.506	14.8°	0.64	0.48
	API–Cobb T12-S1	0.412	−32.5°	1.00	0.02

Note: The sacral base angle (SBA) showed a moderate ability [34] to discriminate between the normal and acute low back pain groups. The maximum value of Youden’s index was used as the criterion for choosing the cut-off. Variables are as follows: lumbar lordosis (ARA L1-L5, ARA T12-S1, and Cobb T12-S1), sacral base angle (SBA), posterior tangent of S1 to the vertical (PT-S1), angle of pelvic incidence (API), posterior tangent pelvic incidence angle (PTPIA), elliptical minor to major axis ratio (b/a), API-ARA T12-S1, API-ARA L1-L5, and API-Cobb T12-S1. A negative rotational value indicates spinal extension.

## Data Availability

Correspondence and requests for materials should be addressed to D.E.H.
